# Deep tissue penetration of nanoparticles using pulsed-high intensity focused ultrasound

**DOI:** 10.1186/s40580-017-0124-z

**Published:** 2017-11-08

**Authors:** Dong Gil You, Hong Yeol Yoon, Sangmin Jeon, Wooram Um, Sejin Son, Jae Hyung Park, Ick Chan Kwon, Kwangmeyung Kim

**Affiliations:** 1 School of Chemical Engineering, Sungkyunkwan University, 2066, Seobu-ro, Jangan-gu, Suwon, 16419 Republic of Korea; 2 Center for Theragnosis, Biomedical Research Institute, Korea Institute of Science and Technology, 5, Hwarang-ro 14-gil, Seongbuk-gu, Seoul, 02792 Republic of Korea; 3 KU-KIST Graduate School of Converging Science and Technology, Korea University, 145 Anam-ro, Seongbuk-gu, Seoul, 02841 Republic of Korea

**Keywords:** Pulsed-high intensity focused ultrasound, Tissue penetration, Nanoparticle, Drug delivery

## Abstract

Recently, ultrasound (US)-based drug delivery strategies have received attention to improve enhanced permeation and retention (EPR) effect-based passive targeting efficiency of nanoparticles in vitro and in vivo conditions. Among the US treatment techniques, pulsed-high intensity focused ultrasound (pHIFU) have specialized for improving tissue penetration of various macromolecules and nanoparticles without irreversible tissue damages. In this study, we have demonstrated that pHIFU could be utilized to improve tissue penetration of fluorescent dye-labeled glycol chitosan nanoparticles (FCNPs) in femoral tissue of mice. pHIFU could improve blood flow of the targeted-blood vessel in femoral tissue. In addition, tissue penetration of FCNPs was specifically increased 5.7-, 8- and 9.3-folds than that of non-treated (0 W pHIFU) femoral tissue, when the femoral tissue was treated with 10, 20 and 50 W of pHIFU, respectively. However, tissue penetration of FCNPs was significantly reduced after 3 h post-pHIFU treatment (50 W). Because overdose (50 W) of pHIFU led to irreversible tissue damages, including the edema and chapped red blood cells. These overall results support that pHIFU treatment can enhance the extravasation and tissue penetration of FCNPs as well as induce irreversible tissue damages. We expect that our results can provide advantages to optimize pHIFU-mediated delivery strategy of nanoparticles for further clinical applications.

## Introduction

Over the past several decades, nanotechnology based drug delivery platforms have emerged as an ‘All-in-one systems’ for diagnosis and therapy of diseases, including cancer. In particular, nanoparticle-based drug delivery systems could improve solubility and circulation time of therapeutic agents, resulting in overcoming pharmacokinetic limitation related with conventional drug formulations [1–3]. Based on the enhanced permeability and retention (EPR) effect, nanoparticles could be accumulated by extravasation through leaky blood vessels in the angiogenesis-related diseases, such as infection, heart failure, renal disease and cancer [4, 5].

Recently, with an increasing pathological evidence of EPR phenomenon, ultrasound (US)-based drug delivery strategies have received attention to improve EPR-based passive targeting efficiency of nanoparticles in vitro and in vivo conditions [6–9]. As an advanced technique of US treatment, high intensity focused ultrasound (HIFU) which can focus US intensity at the target site has developed for ablating various types of live tissue, such as prostate cancer, breast cancer, liver cancer, and fibroid [10–13]. In combination with diagnostic US, magnetic resonance imaging (MRI), and computed tomography (CT), HIFU can specifically exposure to target site, resulting in reducing blood loss, scar formation, and eliminating the risk of infection [14]. Despite the successes application of HIFU, however, the optimization of HIFU treatment is still challenging for improving synergistic effects of nanoparticle-based drug delivery systems due to irreversible thermal effect, including hemostasis and blood vessel occlusion. In this point of view, pulsed-HIFU (pHIFU) treatment can reduce irreversible thermal effects by reducing the average intensity of the target tissue [15, 16]. Furthermore, pHIFU treatment could improve delivery efficiency of theranostic materials to the target sites by an increasing extravasation from the vasculature and improving tissue penetration [17]. In the previous study, we have demonstrated that extracellular matrix (ECM) remodeling and disruption of the collagen matrix through pHIFU treatment for improving the deep tissue penetration and tumor targeting efficiency of nanoparticles [18]. 20 W/cm^2^ of pHIFU treatment did not occur acute tissue damage but increased blood flow, decreased collagen content, resulting in improving penetration and localization of nanoparticles into the tumor tissue. Although pHIFU could enable the remodeling of ECM and destroy the collagen matrix, resulting in deep tissue penetration of nanoparticles, optimization of the pHIFU-mediated extravasation and tissue penetration of nanoparticles was not clear.

**Scheme 1:**
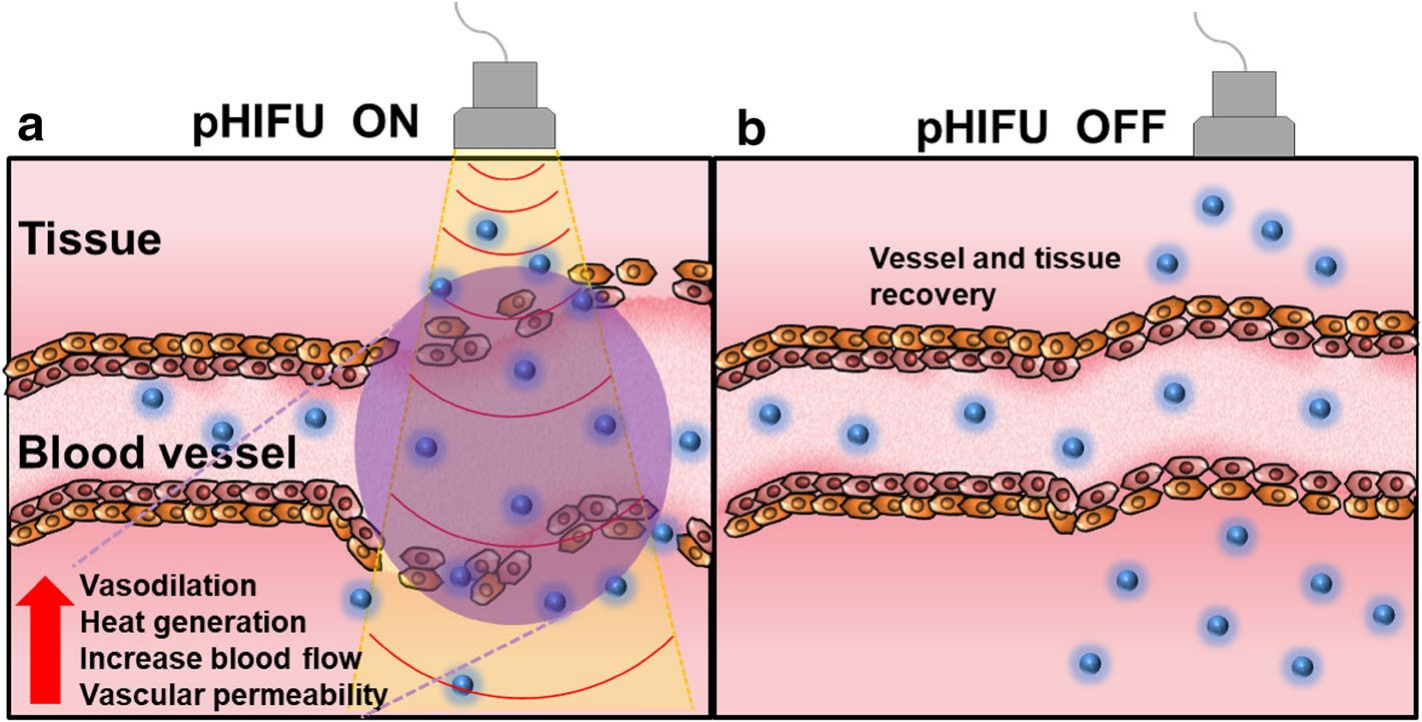
Schematic illustration of pulsed-high intensity focused ultrasound (pHIFU)-mediated tissue penetration enhancement of nanoparticles. **a** The pHIFU-on state allowed to heat-generation, vasodilation effect, and blood flow enhancement in the tissue, resulting in increasing vascular permeability of nanoparticles. **b** The vessels and blood flow in the tissue could recover, when the pHIFU was turned-off

In this study, we observed pHIFU-mediated extravasation effect and enhanced tissue penetration of nanoparticles (Scheme 1a). For these purposes, fluorescent dye modified glycol chitosan nanoparticles (FCNPs) and femoral vein of mice were used as a model nanoparticle and as a model blood vessel, respectively. We observed stability of FCNPs after pHIFU treatment in vitro. And then, pHIFU-mediated blood flow enhancement and hyperthermia in mice femoral tissue were observed using Doppler ultrasound and tissue histological analysis, respectively. Finally, we carefully analyzed pHIFU-mediated extravasation and tissue penetration of FCNPs with the blood vessel recovery in mice femoral vein using non-invasive real time near-infrared fluorescence imaging (Scheme 1b).

## Materials and methods

### Materials

Glycol chitosan (MW = 2.5 × 10^5^ Da; degree of deacetylation = 82.7%), 5β-cholanic acid, anhydrous methanol, dimethyl sulfoxide (DMSO), N-hydroxysuccinimide (NHS), 1-ethyl-3-(3-dimethylaminopropyl)-carbodiimide hydrochloride (EDC) and Trichrome Stain (Masson) kit were purchased from Sigma-Aldrich (St. Louis, MO, USA). Near infrared fluorescence (NIRF) dye Flamma^®^ 675 NHS ester was purchased from BioActs (Incheon, Korea). Perfluoropentane (PFP, 99%) was purchased from Apollo Scientific Ltd. (Manchester, UK). All other chemicals were analytical grade and used without further purification.

### Preparation of hydrophobically modified glycol chitosan nanoparticle (CNPs) and fluorescent dye labeled CNPs (FCNPs)

The amphiphilic glycol chitosan conjugate was synthesized by conjugation of 5β-cholanic acid to the glycol chitosan backbone [19]. In brief, glycol chitosan (500 mg, 2 µmol) was dissolved in distilled water/methanol [125 ml, 1:1, (v/v)]. Glycol chitosan solution was mixed with EDC (120 mg, 625 µmol) and NHS (72 mg, 625 µmol) for 30 min at 25 °C and then 125 ml of methanol was added to glycol chitosan solution. The glycol chitosan solution was drop-wised to 5β-cholanic acid (150 mg, 416 µmol) containing methanol (125 ml). The reaction mixture was stirred for 24 h at 25 °C. The resulting solution was dialyzed against methanol/distilled water (1:0, 1:1, 0:1, v/v) using a dialysis membrane (Spectra/Por^®^6 MWCO 12–14 kDa) for 3 days. Finally, the product was lyophilized to obtain glycol chitosan-5β-cholanic acid conjugate, CNPs. For the near-infrared fluorescence (NIRF) imaging, CNPs were labeled with NIRF dye, Flamma^®^ 675 (λ_ex_ = 675 nm, λ_em_ = 720 nm). In brief, 100 mg of CNPs was dissolved in distilled water/dimethyl sulfoxide (DMSO) (1:1 v/v) and 1 mg of Flamma^®^ 675 NHS ester was dissolved in 100 µl of DMSO. The CNPs solution was mixed with Flamma^®^ 675 NHS ester solution and then the mixture was stirred for 24 h at 25 °C. The resulting solution was dialyzed against distilled water using a dialysis membrane (Spectra/Por^®^6 MWCO 12–14 kDa) for 2 days. Finally, the product was lyophilized to obtain Flamma^®^ 675-labeled CNPs, FCNPs.

### Characterization of FCNPs

**Fig. 1:**
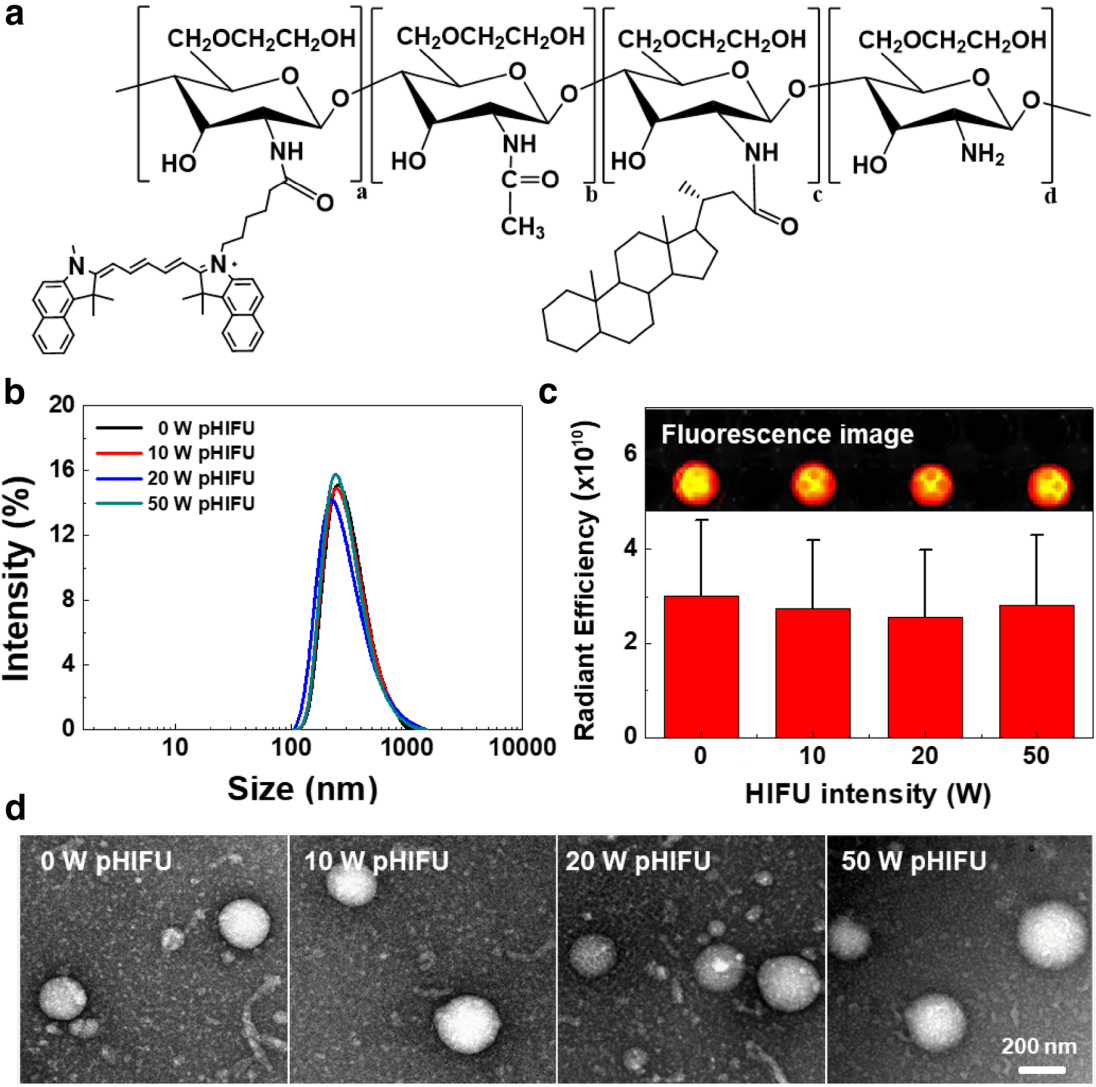
In vitro physicochemical properties of fluorescent dye-labeled glycol chitosan nanoparticles (FCNPs). **a** Structure of FCNPs. **b** Hydrodynamic size distribution of FCNPs after treatment of 10, 20, and 50 W pHIFU. **c** Fluorescence stability of FCNPs after treatment of 10, 20, and 50 W pHIFU. **d** Transmission electron microscope (TEM) images of FCNPs after treatment of 10, 20, and 50 W pHIFU. Scale bar indicates 200 nm

To confirm the deformation and fluorescence stability of FCNPs against pHIFU treatment, the hydrodynamic size distribution of FCNPs was measured using dynamic laser scattering (DLS) after pHIFU treatment. In brief, 10 mg of FCNPs was dispersed in 10 ml of distilled water (1 mg/ml) and FCNPs solution was transferred into agarose mold for pHIFU treatment. For the evaluation of deformation of FCNPs by pHIFU treatment, the hydrodynamic size distribution of FCNPs was measured by DLS (Nano ZS, Malvern, UK) after 10, 20 and 50 W (W)-pHIFU treatment (Frequency 1.5 MHz, Duty cycle 10%, Pulse repetition frequency 1 Hz, Time 30 s, Interval 2 mm) (Fig. 1b). The fluorescence stability of pHIFU-treated FCNPs was determined using the near-infrared fluorescence (NIRF) imaging system (IVIS Lumina Series III, PerkinElmer, Massachusetts, USA). Fluorescence intensities from pHIFU-treated FCNPs were analyzed using the Living Image^®^ software (PerkinElmer, Massachusetts, USA) (Fig. 1c). The morphology of FCNPs was observed by transmission electron microscope (TEM, Tecnai F20, FEI, Netherlands) at an accelerating voltage of 200 ekV. For TEM images, all samples were dispersed in distilled water and were negative stained by 2% uranyl acetate (Fig. 1d).

### Observation of pHIFU-mediated blood flow enhancement using Doppler ultrasound

All experiments with live animals were performed in compliance with the relevant laws and institutional guidelines of Institutional Animal Care and Use Committee (IACUC) in Korea Institute of Science and Technology (KIST), and IACUC approved the experiment (Approved Number of 2017-005). To observation of enhancement blood flow by pHIFU treatment in vivo, BALB/c nude mouse (5 week-old, 19–25 g, male) was purchased from Nara Bio INC. (Gyeonggi-do, Korea). pHIFU-mediated blood flow enhancement was observed by Doppler ultrasound (ECUBE-7, Alpinion, Korea) with Color-flow (CF mode) and Pulsed-wave (PW mode) modes at the femoral vessel region of mice after treatment of 10, 20 and 50 W pHIFU.

#### pHIFU treatment

Frequency 1.5 MHz, Duty cycle 10%, Pulse repetition frequency 1 Hz, Time 30 s, Interval 2 mm.

#### CF mode

Freqeuncy: 4.5 MHz, Pulse repetition frequency: 3.6 kHz, Threshold: 80.

#### PW mode

Frequency: 4.5 MHz, Pulse repetition frequency: 2.6 kHz, Sweep: 0.

### Observation of pHIFU-induced hyperthermia

To observe pHIFU-induced hyperthermia, 50 μm of thermocouple wire (Physitemp Instrument Inc., Clifton, USA) was directly inserted into femoral tissue of BALB/c nude mice. The temperature of femoral tissue was monitored after 10, 20 and 50 W pHIFU treatment (Frequency 1.5 MHz, Duty cycle 10%, Pulse repetition frequency 1 Hz, Time 30 s, Interval 2 mm) (Fig. 3a). To evaluate tissue damage after pHIFU-treatment, femoral tissue was dissected from the mice. And then, the femoral tissue was fixed with 4% paraformaldehyde solution and embedded in paraffin. The sliced organs (6 μm) were stained by Masson’s trichrome (MT) staining protocol and observed by optical microscope (BX 51, Olympus, USA) (Fig. 3b).

### Observation of pHIFU-mediated tissue penetration of FCNPs using in vivo NIRF imaging system

To observe pHIFU-mediated tissue penetration of FCNPs, 5 mg/kg of FCNPs were intravenously injected into the BALB/c nude mice and then 10, 20 and 50 W pHIFU were treated to the femoral tissue. The pHIFU-mediated tissue penetration effect was evaluated using time-dependent fluorescence images at the femoral tissue which obtained by a Small Animal Imaging System (OV-100, Olympus, Japan) (Fig. 4a). For the visualization of vessels, 20 mg/kg of fluorescein isothiocyanate (FITC)-labeled dextran was intravenously injected into the mice. The fluorescence of vessels and FCNPs were acquired using GFP channel (λ_ex_ = 450−480 nm, λ_em_ = 500-530 nm, exposure time = 100 ms, offset value = 100) and Cy5.5 channel (λ_ex_ = 620-650 nm, λ_em_ = 680-710 nm, exposure time = 6000 ms, offset value = 200), respectively. The fluorescence intensities from a femoral tissue were analyzed by Image-pro plus software (Media Cybernetics, USA) (Fig. 4b).

### In vivo tissue penetration recovery analysis in pHIFU-treated femoral tissue

To observe vessel and tissue penetration recovery after pHIFU-treatment, the femoral tissue of the BALB/c nude mice was treated with 20 and 50 W pHIFU. And then the femoral tissue was rested for 0, 1 and 3 h for the tissue structure recovery. Subsequently, 200 μl of FCNPs (5 mg/kg) was intravenously injected into the mice (n = 4) and the fluorescence from FCNPs in femoral tissue was acquired at 30 min post-injection of FCNPs by a Small Animal Imaging System with Cy5.5 channel (λ_ex_ = 620–650 nm, λ_em_ = 680–710 nm, exposure time = 6000 ms, offset value = 200). The fluorescence intensities from a femoral tissue were analyzed by Image-pro plus software (Media Cybernetics, USA). The vessel and tissue penetration recovery effect were evaluated by the relative fluorescence intensity of between non-treated (0 W pHIFU) tissue and pHIFU-treated tissue (Fig. 5c).

### Statistical analysis

In this study, the differences between experimental and control groups were analyzed using one-way ANOVA and considered statistically significant (marked in figure).

## Results and discussion

### Preparation and characterization of fluorescent dye labeled CNPs (FCNPs)

To observe tissue penetration of nanoparticles in the mice model, fluorescent dye labeled glycol chitosan nanoparticle (FCNPs) was used as a model nanoparticle. We already confirmed that CNPs were formed stable nanoparticle in blood flow, and they had enough deformability to extravasation in vivo [20, 21]. FCNPs was prepared by chemical conjugation of 5β-cholanic acid and fluorescent dye to glycol chitosan polymer (Fig. 1a). FCNPs formed nano-sized particle, which was 290 ± 8.3 nm under the aqueous condition. This is because FCNPs have amphiphilic property based on the hydrophobic 5β-cholanic acid and the hydrophilic glycol chitosan polymer [22]. The size and fluorescence stability of FCNPs were evaluated after 10, 20 and 50 W of pHIFU treatment. The size of 0, 10, 20 and 50 W pHIFU-treated FCNPs were measured 298.4 ± 13.36, 300.8 ± 8.66, 308.3 ± 9.99 and 314.9 ± 13.43 nm, respectively. Furthermore, the size distribution of FCNPs showed only negligible changes after pHIFU treatment compared to that of non-treated (0 W pHIFU) FCNPs (Fig. 1b). This is because FCNPs stable nanoparticle in the aqueous condition, and they did not dissociate by pHIFU treatment. Fluorescence images of FCNPs did not show significant changes after pHIFU treatment compared to that of non-treated (0 W pHIFU) FCNPs (Fig. 1c). The TEM images showed that FCNPs could form spherical nanoparticle in the aqueous condition, and the morphology did not change by pHIFU treatment (Fig. 1d). Therefore, we expected that the stability of FCNPs against pHIFU was enough to visualization of pHIFU-mediated extravasation and tissue penetration in vivo.

### Observation of pHIFU-mediated blood flow enhancement using the Doppler ultrasound

**Fig. 2:**
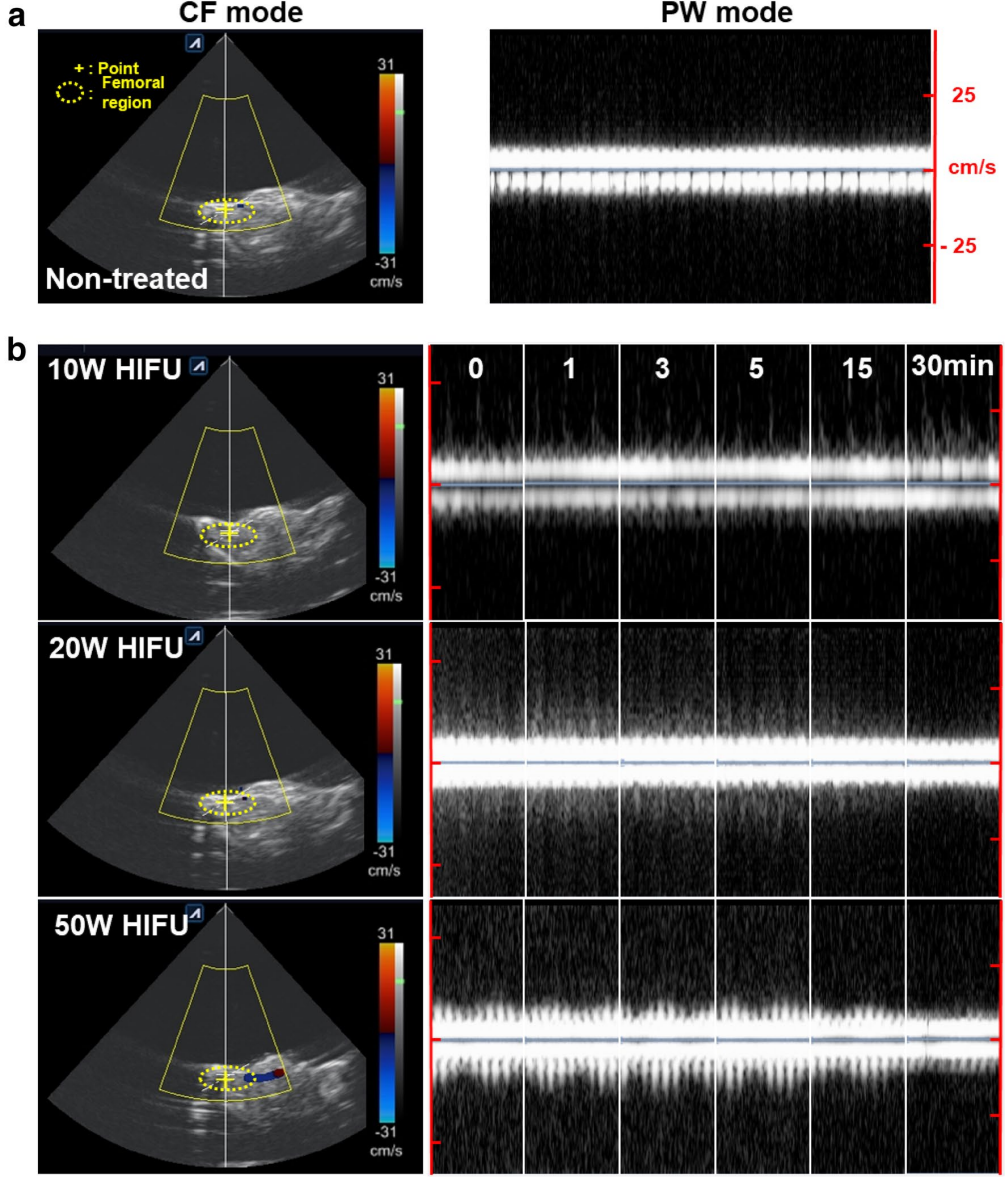
pHIFU-mediated blood flow enhancement. Color-flow Doppler ultrasound image and pulsed-wave Doppler image of **a** non-treated (0 W pHIFU) and **b** 10, 20, and 50 W pHIFU-treated femoral tissue. Yellow dot circles indicate the femoral vessel region

pHIFU-mediated blood flow enhancement was observed using the Doppler ultrasound imaging. The Doppler ultrasound is a non-invasive real-time pulse-wave technique, and it can utilize in monitoring hemodynamics in vivo. The color flow (CF) mode and pulsed wave (PW) mode of Doppler ultrasound images of the femoral tissue did not show blood flow enhancement without pHIFU treatment (Fig. 2a). However, CF signals and PW signals were gradually increased after 10, 20 and 50 W pHIFU treatment, indicating that the velocity of blood flow was increased by the pHIFU treatment energy (Fig. 2b). Interestingly, 10, 20 and 50 W pHIFU-treated femoral tissue showed that the width of Doppler signal was increased within 5 min after pHIFU treatment. However, the width of Doppler signal was rapidly reduced after 50 W pHIFU treatment for 15 min, indicating that the blood flow was reduced in femoral tissue.

### Observation of pHIFU-induced hyperthermia

**Fig. 3:**
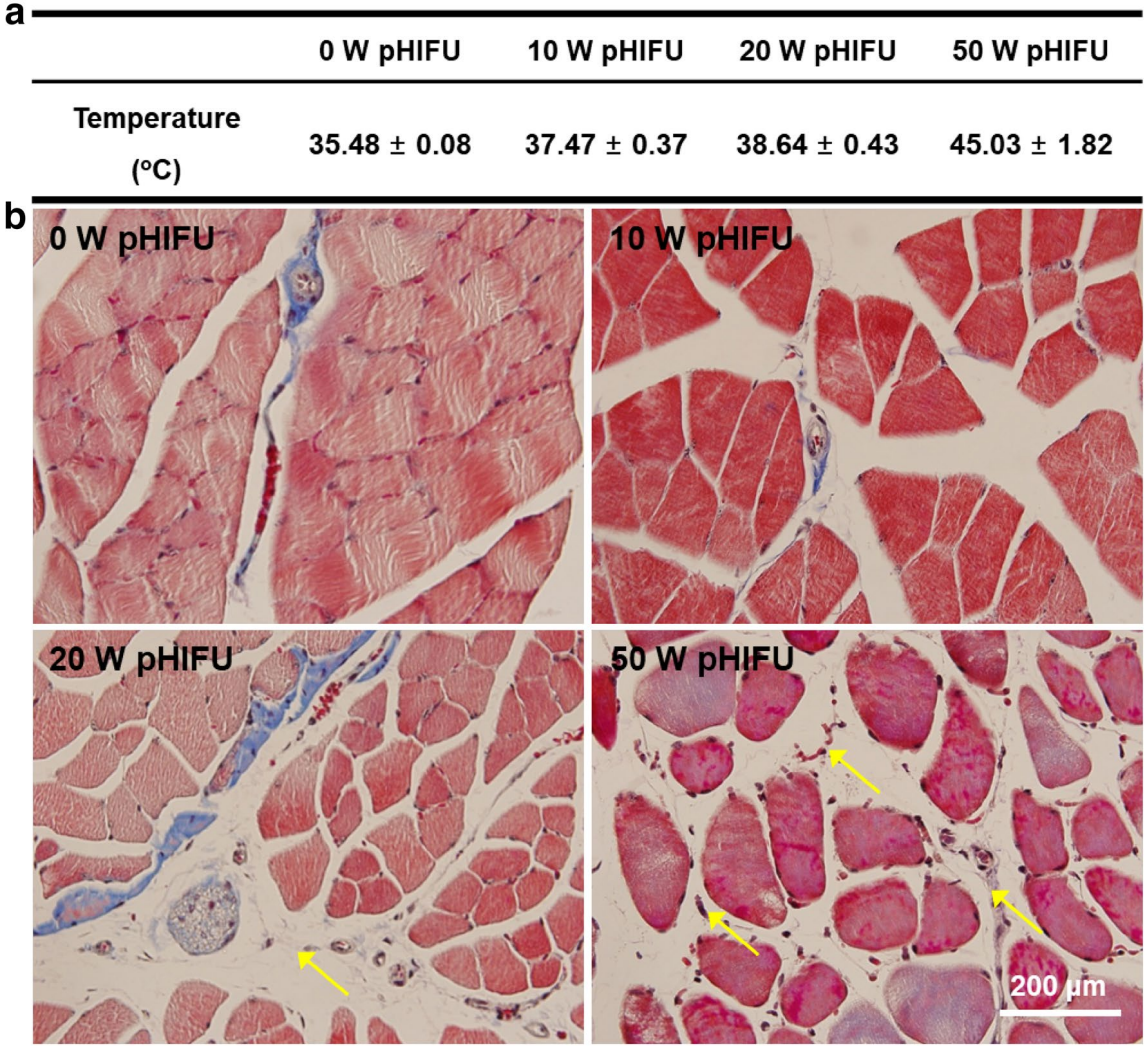
pHIFU-induced hyperthermia effect in femoral tissues. **a** The temperature at femoral tissues after treatment of 10, 20, and 50 W pHIFU. **b** Masson’s trichrome (MT) staining images of femoral tissues after treatment of 10, 20, and 50 W pHIFU. Scale bar indicates 200 µm. Yellow arrows indicate the histopathological abnormalities, such as the edema and chapped red blood cells

Hyperthermia effect by pHIFU treatment can loosen tissue junction in the body. However, repetition and excessive pHIFU-treatment can cause destruction of tissue structures, including vessel and muscle. To confirm the pHIFU-induced hyperthermia, we monitored the temperature in femoral tissue after 10, 20 and 50 W of pHIFU treatments (Fig. 3a). The temperature in femoral tissues was increased to 37.47 ± 0.37, 38.64 ± 0.43 and 45.03 ± 1.82 °C after 10, 20 and 50 W pHIFU treatment, respectively. To observe the histological changes by HIFU-treatment, we performed Masson’s trichrome (MT) staining of femoral tissue after pHIFU treatment (Fig. 3b). The histological structure of non-treated (0 W pHIFU) femoral tissue showed tight junctions in muscle whereas leaky junctions in muscle were observed in 10, 20 and 50 W pHIFU-treated femoral tissue. The junction deformation effect was enhanced by an increment of pHIFU-treatment power. However, the MT staining images showed leaky junction in muscle as well as the edema and chapped red blood cells wherein 20 and 50 W pHIFU-treated femoral tissue. This is because 20 and 50 W of pHIFU treatment allow for significant elevations in temperature, resulting in occurring irreversible thermal effects in femoral tissue.

### Observation of pHIFU-mediated tissue penetration of FCNPs using in vivo NIRF imaging system

**Fig. 4:**
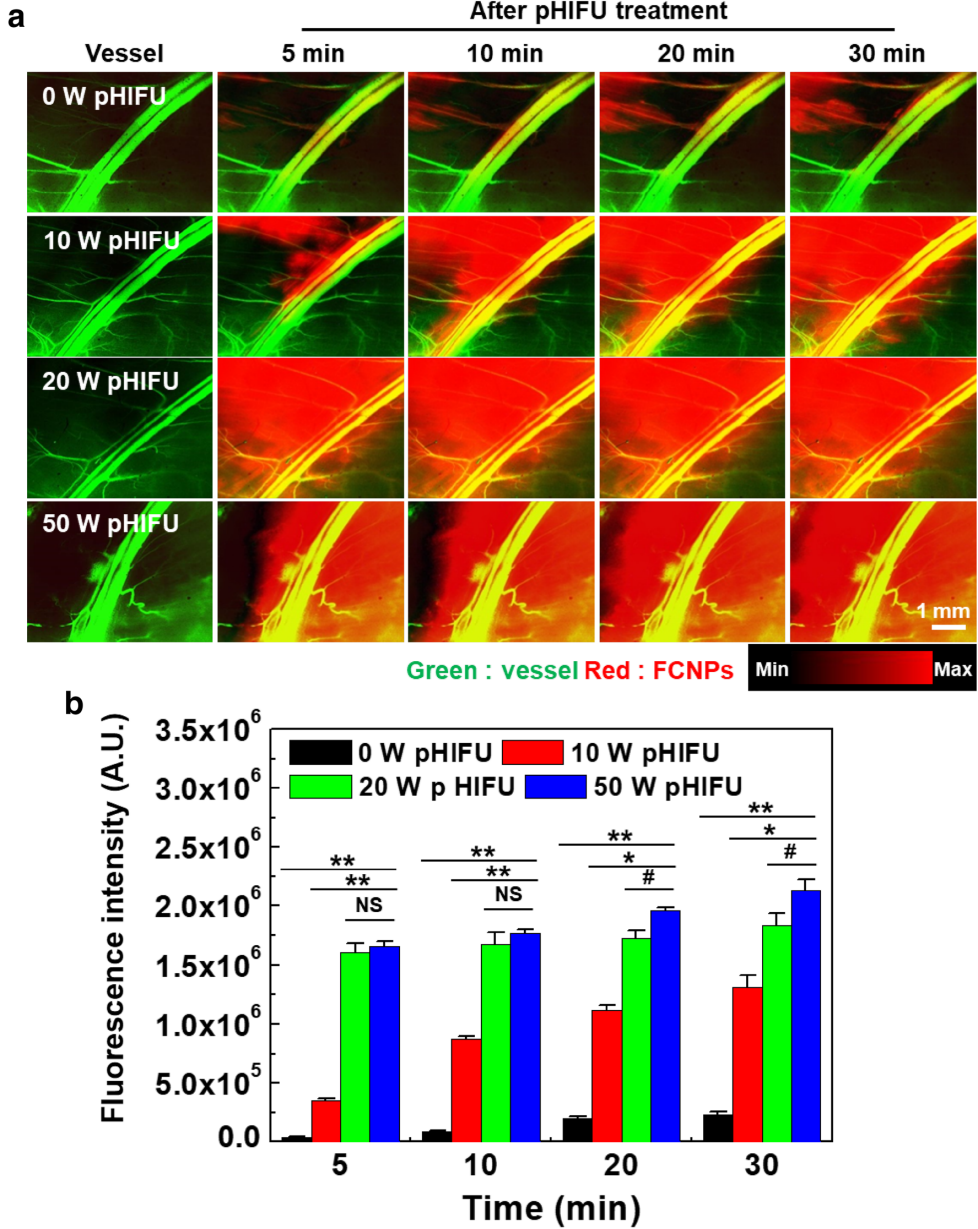
pHIFU-mediated tissue penetration effect of FCNPs using in vivo NIRF imaging system. **a** Fluorescence images of femoral tissues, which intravenously injected FCNPs followed by 10, 20, and 50 W pHIFU treatment. Scale bar indicates 1 mm. **b** Quantified fluorescence intensity of FCNPs in femoral tissues. ^#^, *, and ** indicate difference at the *p* < 0.05, *p* < 0.01, and *p* < 0.001 significance level, respectively

To monitor pHIFU-mediated tissue penetration effect of FCNPs, 5 mg/kg of FCNPs was intravenously injected to the mice. And then, the fluorescence signals from FCNPs in femoral tissue were monitored at 5, 10, 20 and 30 min during 10, 20 and 50 W of pHIFU treatment (Fig. 4a). The fluorescence signals from FCNPs were gradually increased for 30 min in the femoral tissue when the femoral tissue was treated with 10 W of pHIFU. Furthermore, strong fluorescence signals in the femoral tissue could be observed when it treated with 20 and 50 W of pHIFU. However, the fluorescence signal in femoral tissue was slightly increased for 30 min without pHIFU treatment, indicating that FCNPs could rarely penetrate the vessel and diffuse into the femoral tissue. Finally, the fluorescence intensities of FCNPs which was treated with 10, 20 and 50 W of pHIFU for 30 min were 5.7-, 8- and 9.3-folds higher than that of non-treated (0 W pHIFU) femoral tissue, respectively (Fig. 4b).

### In vivo tissue penetration recovery analysis in pHIFU-treated femoral tissue

**Fig. 5:**
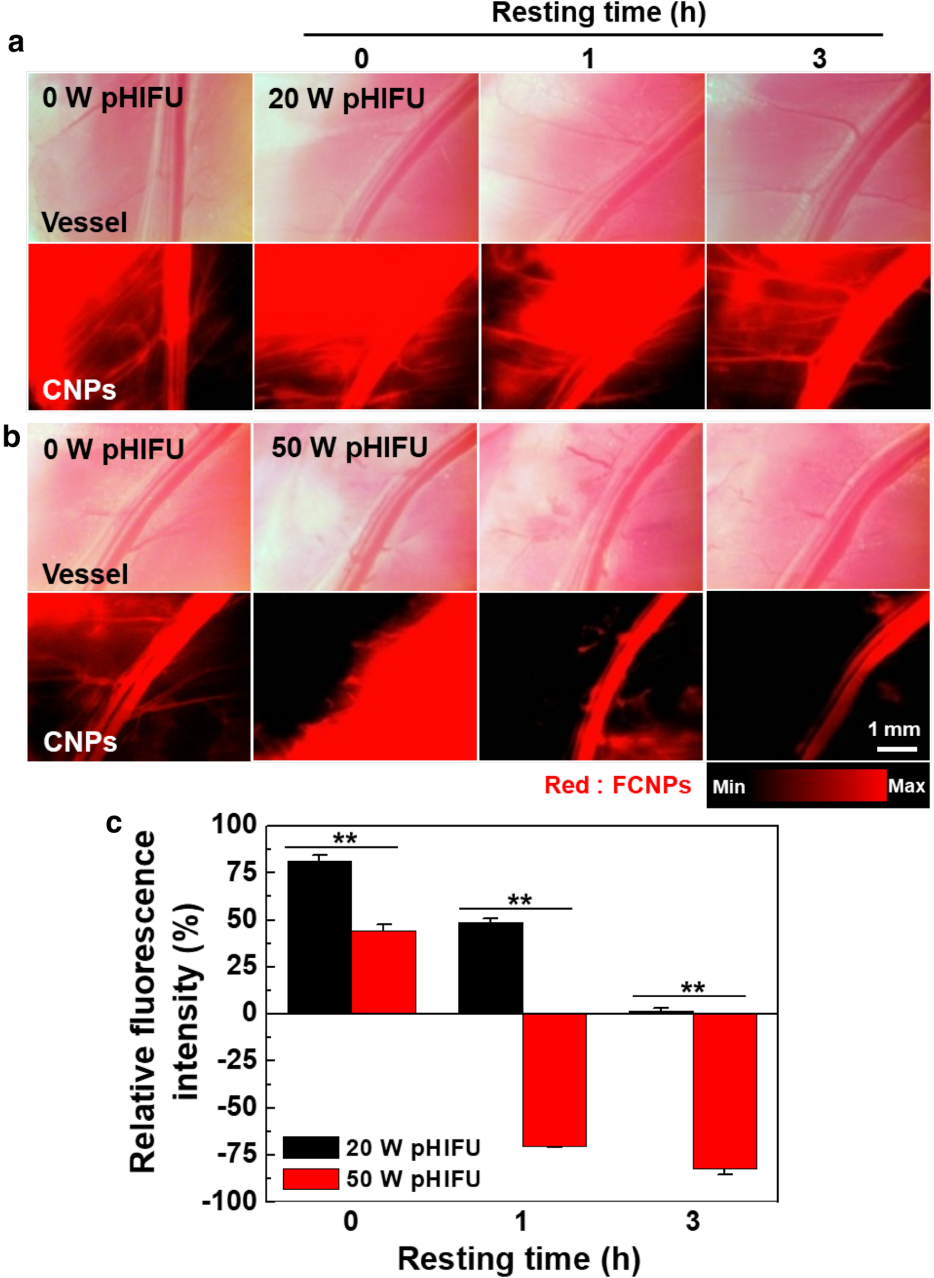
In vivo tissue penetration recovery analysis in pHIFU-treated femoral tissue. **a, b** Fluorescence images of femoral tissues, which rested for 0, 1, and 3 h after treatment of 0, 20, and 50 W pHIFU. The fluorescence signal was monitored at 30 min after intravenously injecting of FCNPs. Scale bar indicates 1 mm. **c** Relative fluorescence intensity of FCNPs in femoral tissues. ** indicates difference at the *p* < 0.001 significance level

The tissue penetration of FCNPs could be reduced due to the irreversible damages in vessel and peripheral tissues by a pHIFU treatment. Thus, optimization of pHIFU-treatment condition is important for improving delivery efficiency of nanoparticles or drugs to target tissue. In this point of view, we evaluated tissue penetration of FCNPs by a Small Animal Imaging System after 20 and 50 W of pHIFU treatment. To recover tissue properties, pHIFU-treated femoral tissue was allowed to rest for 0, 1 and 3 h. And then, the fluorescence signal in femoral tissue was observed at 30 min after intravenously injecting of FCNPs (5 mg/kg). As shown in Fig. 5a, the fluorescence signal of FNCPs in the 1 and 3 h-rested femoral tissue was gradually decreased as that of non-treated femoral tissue, when they were treated with 20 W of pHIFU. In addition, the fluorescence signal could observe not only vessels but also peripheral tissues after 1 and 3 h resting. However, the fluorescence signal of FCNPs could only observe in vessels after 1 and 3 h resting, when the femoral tissues were treated with 50 W of pHIFU (Fig. 5b). As shown in Fig. 5c, when the FCNPs were subsequently injected after 20 and 50 W pHIFU treatment (0 h), the relative fluorescence intensity of both 20 and 50 W pHIFU-treated femoral tissue was 81.2 and 44.1% higher than that of non-treated (0 W pHIFU) femoral tissue, respectively. This is because that vessel and tissue penetration of FCNPs were enhanced by pHIFU treatment. In addition, the relative fluorescence intensity in 1 and 3 h-rested femoral tissues was gradually decreased to 49 and 1.6% of that of non-treated femoral tissue, indicating that the vessel and tissue could be recovered to that of the before pHIFU treatment. On the other hand, the relative fluorescence intensity in 1 and 3 h-rested femoral tissues was dramatically changed which was − 70 and − 82.1% compared to that of non-treated (0 W pHIFU) femoral tissue, respectively. This is because that the blood vessels in femoral tissue which could utilize to delivery route of FCNPs was damaged by 50 W of pHIFU treatment, resulting in reducing tissue penetration of FCNPs.

## Conclusions

In summary, we have optimized treatment method (Frequency 1.5 MHz, Duty cycle 10%, Pulse repetition frequency 1 Hz, Time 30 sec, Interval 2 mm) and demonstrated that we are able to use pulsed-HIFU (pHIFU) to enhance extravasation and tissue penetration of nanoparticles in femoral tissue of mice. pHIFU could improve blood flow of the targeted-blood vessel in femoral tissue. Notably, the extravasation and tissue penetration of FCNPs in femoral tissue was significantly increased by the subsequent treatment of pHIFU. Therefore, pHIFU can be a promising technique that improves delivery efficiency of nanoparticles and overcomes the current limitations of nanoparticle delivery, tissue penetration. However, pHIFU could induce hyperthermia into the target tissue by an increasing the treatment power, resulting in occurring histopathological abnormalities in the target tissue. In addition, overdose treatment of pHIFU could lead to irreversible tissue damages, resulting in reducing the tissue penetration of nanoparticles. Thus, treatment dose of pHIFU should be optimized for further clinical application of pHIFU-based drug delivery strategies.
